# Reconceiving Reproductive Health Systems: Caring for Trans, Nonbinary, and Gender-Expansive People During Pregnancy and Childbirth

**DOI:** 10.1017/jme.2022.88

**Published:** 2022

**Authors:** Elizabeth Kukura

**Affiliations:** 1. DREXEL UNIVERSITY, PHILADELPHIA, PA, USA

**Keywords:** Transgender, Nonbinary, Pregnancy, Childbirth, Reproductive Health, Midwifery

## Abstract

This article examines the barriers to quality health care for transgender, nonbinary, and gender-expansive people (TGE) who become pregnant and give birth, identifying three central themes that emerge from the literature. These insights suggest that significant reform will be necessary to ensure access to safe, appropriate, gender-affirming care for childbearing TGE people. After illustrating the need for systemic changes that untether rigid gender norms from the provision of perinatal care, the article proposes that the Midwives Model of Care offers a set of values and clinical practices that are well-suited to meet the needs of many TGE patients during pregnancy and childbirth and which should be incorporated into the healthcare system more broadly.

Although public discourse about the maternal health crisis in the United States has become more prominent and nuanced in recent years, conversations about poor perinatal health outcomes and their disproportionate impact on communities of color generally ignore the fact that not all people who give birth are women.^1^ Recently, a growing number of transgender men and nonbinary people have come out about their pregnancy and childbirth experiences, challenging the strong association of childbearing with femaleness and unraveling gender stereotypes about pregnancy and birth. For many transgender, nonbinary, and gender-expansive (TGE) people, the best sources of information about conception, accessing care, and dealing with lactation and chestfeeding have been informal networks of people with similar experiences who are willing to share their own childbearing journeys, as research on the pregnancy intentions, experiences, and outcomes of TGE people is quite limited.^2^ However, the number of studies published in the last two years alone reflects a growing body of knowledge about the childbearing experiences of TGE people, as scholars move beyond questions asked in some early literature about *whether* trans people should get pregnant to focus instead on how to best meet the needs of TGE people during pregnancy and childbirth.^3^

This article focuses on the barriers to quality health care for TGE people whose sex assigned at birth was female and who become pregnant and give birth. To date, there is minimal research on the fertility and reproductive experiences of TGE people whose sex assigned at birth was male, though surveys indicate that transgender women want to become parents at the same rate as other LGBTQ+ people.^4^ Focusing here on the childbearing experiences of TGE people who have the capacity for pregnancy, the article will summarize key findings from the existing research on TGE childbirth experiences, identifying three central themes that emerge from the literature. First, research reveals wide diversity in the childbearing experiences of TGE people, highlighting the need for individualized care that accounts for how TGE patients understand their own bodies, relate to broader cultural expectations of pregnancy, and transition into parenthood — whether understood as motherhood, fatherhood, or something else. TGE childbearing experiences vary significantly, and a one-size-fits-all approach to care cannot satisfy such diverse patient needs.^5^ Second, trans, nonbinary, and other gender-expansive people experience both institutional and individual erasure, where the systems that mediate access to perinatal health care and many individual healthcare providers cannot make sense of a pregnant patient who is not a woman and thus treat the patient as female regardless of their actual identity.^6^ Relatedly, the third theme that emerges from research on TGE childbearing experiences is the harm caused by deeply entrenched gender norms surrounding pregnancy and childbirth, which increase the burdens on TGE people when managing their fertility, sustaining healthy pregnancies, and accessing care during labor and delivery.

This article focuses on the barriers to quality health care for TGE people whose sex assigned at birth was female and who become pregnant and give birth. To date, there is minimal research on the fertility and reproductive experiences of TGE people whose sex assigned at birth was male, though surveys indicate that transgender women want to become parents at the same rate as other LGBTQ+ people. Focusing here on the childbearing experiences of TGE people who have the capacity for pregnancy, the article will summarize key findings from the existing research on TGE childbirth experiences, identifying three central themes that emerge from the literature.

Ultimately, these insights suggest that significant reform will be necessary to ensure access to safe, appropriate, gender-affirming care for childbearing TGE people. After illustrating the need for systemic changes that untether rigid gender norms from the provision of perinatal care, the article proposes that the Midwives Model of Care offers a set of values and clinical practices that are well-suited to meet the needs of many childbearing TGE people and which should be incorporated into the healthcare system more broadly. The article concludes by highlighting areas for future research to ensure that the healthcare system evolves to incorporate the needs of this patient population by design, rather than as an exception to an otherwise highly gendered clinical environment.

## I. Defining Terms and Making the Invisible Visible

Transgender (or trans) is a term used to describe people whose gender identity is different from the gender traditionally associated with the sex they were assigned at birth, while cisgender refers to people whose gender identity aligns with their sex assigned at birth.^7^ In this context, sex refers to a person’s anatomical and physiological characteristics, while gender refers to socially constructed identities influenced by roles, norms, and behaviors that society associates with either male or female sex.^8^ Gender dysphoria is a term used to describe clinically significant stress or impairment related to an incongruence between one’s gender and sex assigned at birth; it is recognized by the Diagnostic and Statistical Manual of Mental Disorders to guide clinical diagnoses, though the term is also used in non-clinical contexts to describe the negative impact of being misgendered or otherwise unable to live fully in one’s gender identity.^9^

Someone whose gender identity does not align with their sex assigned at birth may change their physical appearance, name and pronouns, and other attributes in order to live outwardly in a manner that reflects their gender identity — a process referred to as social transitioning. In addition, some people seek gender-affirming medical care, including hormones or surgery, to reduce the discordance they feel between their physical bodies and their gender identity. Historically, many jurisdictions that legally recognize a change in gender have required individuals to undergo medical transition, including sterilization, often at great expense and regardless of the wishes of the transitioning individual.^10^ More recently, some of those jurisdictions have eliminated surgical requirements, allowing for greater diversity in how individuals pursue personally meaningful gender-affirming care, though globally, legal recognition of a change in gender remains a burdensome process or entirely unavailable.^11^

Not all people whose gender differs from their sex assigned at birth experience gender as one of only two options. Nonbinary is a term adopted by some people whose identity falls outside a gender binary; other terms include genderqueer, gender fluid, or gender non-conforming. Nonbinary people may or may not use medical approaches to align their identities and physical bodies, and, like transgender people, some nonbinary people experience gender dysphoria while others do not.

In the United States, approximately 1.4 million adults — or 0.6% of the population — identify as transgender.^12^ In addition, 1.2 million LGBTQ people in the United States identify as nonbinary, the majority of whom identify as cisgender rather than transgender.^13^ The challenges transgender and nonbinary people encounter accessing appropriate perinatal care are not identical, a reflection of how gender norms — and particularly, a commitment to the gender binary — shape this healthcare specialty. Existing research focuses predominantly on transgender experiences to the exclusion of nonbinary and other gender diverse people.^14^ Following the approach of leading researchers in the field of transgender health, this article uses the terms transgender, nonbinary, and gender-expansive, along with the acronym “TGE,” to be inclusive when discussing the healthcare system’s failures to provide appropriate perinatal care for gender diverse people, though discussion of particular research findings will reflect the relevant terminology when studies focused exclusively on trans male experiences.^15^

There are no reliable data on the number of TGE people who become pregnant or give birth in the United States, but anecdotal observations indicate that pregnancy among trans men has increased in frequency and visibility in recent years.^16^ Some commentators suggest that the easing of surgical requirements for legal recognition of post-transition gender has enabled TGE people to fulfill parenting wishes that had previously been foreclosed by the rigid framework for administrative acknowledgment of a change in gender.^17^ More generally, surgical requirements for gender recognition tend to reinforce a false perception of trans people as unlikely to pursue parenthood through pregnancy or to become pregnant unexpectedly.^18^ This is slowly changing, along with an increasing number of people who transition at younger ages — all of which has increased the “legibility of being pregnant and male” and made TGE childbearing a less lonely and isolating experience.^19^ Nevertheless, despite the “optimism and hope” about prospects for childbearing reported by some TGE study participants, significant barriers remain to accessing appropriate care during pregnancy and childbirth.^20^

## II. Surveying the Research

In the extensive research literature on pregnancy and childbirth, the experiences of TGE people have been largely ignored. Researchers ask questions and use language that foreclose the possibility of pregnancy among individuals who are not cisgender women. But a small number of studies challenge the hegemonic association of childbearing with cisgender women, revealing areas where the healthcare system often fails TGE people during pregnancy and childbirth, as well as highlighting factors that contribute to positive birth experiences for TGE people. This research suggests that anywhere from 24% to 58% of TGE people want to have children, a broad range that illustrates the urgency of collecting better data and using such data to integrate the needs of TGE patients into provision of perinatal care services.^21^ Importantly, racial and ethnic minorities, people living on low incomes, and people with disabilities are underrepresented in the existing literature, so the ability to draw conclusions is limited by small sample sizes that do not reflect the full diversity of the TGE population.^22^

Rather than summarize the research literature on TGE childbearing as a whole, this section will highlight key findings against the backdrop of three interconnected themes: (1) the diversity of TGE childbearing experiences; (2) the institutional and individual erasure of TGE people within perinatal health care; and (3) the harm caused by entrenched gender norms in the provision of childbirth-related care.

### A. Diversity of TGE Childbearing Experiences

Research on pregnancy and childbirth among TGE people reveals a wide range of experiences in becoming pregnant, navigating pregnancy, managing gender transition and pregnancy, giving birth, and infant feeding. Such diversity is reflected in the array of terms participants use to describe their own gender, including “male,” “man,” “female-to-male,” “transman,” “trans man,” “transgender man,” “transmasculine,” “nonbinary,” and “on the transmasculine spectrum.”^23^ Some participants referred to themselves as fathers or mothers, while others preferred a neutral term like parent to avoid dysphoria associated with gender-specific language.^24^ Variation in language extends to discussion of body parts. For example, some people prefer “chest” over “breast” or “front hole” or “front pelvic opening” over “vagina,” leading experts to recommend that providers invite patients to complete a body part self-inventory and then mirror the language used by the patient.^25^

#### 1. Conception

Studies report that TGE people become pregnant by various means and with varying degrees of intention and enthusiasm, similar to cisgender people. For some, pregnancy was “strongly desired,” while others considered it a “tolerable means to become a parent” and still others became pregnant unexpectedly.^26^ One study reported that approximately 30% of transgender men experienced unexpected pregnancies.^27^ While research indicates similar rates of contraception use between the TGE population and the national average (60% v. 62%), there may be differences in preferred method among TGE people depending on whether someone has taken testosterone.^28^ For example, one study showed trans men who had taken testosterone were more likely to use an IUD than people who had never used testosterone, though IUD insertion itself may trigger dysphoria in some patients.^29^

For intended pregnancies, it is common for TGE people to use their own eggs, while participants reported securing sperm from a variety of sources: their committed partners, sexual partners in the absence of a long-term relationship, known donors, and anonymous donors.^30^ Given the limited number of studies and their small sample sizes, it is impossible to draw meaningful conclusions about how age, race and ethnicity, socioeconomic status, and disability relate to TGE people’s decision-making about conception.

Among TGE people who become pregnant unexpectedly, some continue the pregnancy while others choose to terminate. Analysis of the many barriers to abortion access across the United States is beyond the scope of this article, but the limited research on abortion experiences or preferences of TGE people suggests some overlap between issues of concern to TGE abortion patients and the problems encountered by TGE pregnant people seeking perinatal health care, including highly gendered clinical spaces, systems that exclude the possibility of non-female patients seeking care, the difficulty of accessing affirming care, and the need for greater patient privacy.^31^

#### 2. Navigating the World as Pregnant

There is no typical experience for TGE people as they navigate the world during nine months of pregnancy. Some report living visibly as out pregnant trans men. Doing so affirmed both their gender identity and their pregnancy but increased fears of hostility and discrimination.^32^ Others continued to present as male but not as pregnant, often being “perceived as a fat man.”^33^ Feeling visible as a man but invisible as pregnant decreased the violence and transphobia participants experienced, but such individuals missed opportunities for social support and affirmation regarding their pregnancies.^34^ Still other TGE people report passing as cisgender women during their pregnancies.^35^ Researchers reported that pregnant TGE people passing as cisgender women enjoyed the benefits (such as social support and affirmation) and empowerment associated with being legible as pregnant in public, while feeling safer and experiencing less overt transphobia, though this experience was often accompanied by increased gender dysphoria.^36^ In general, study participants vary in how much they value being seen as pregnant. While it was very important for some people to be recognized as pregnant, others preferred that the pregnancy not be publicly acknowledged and prioritized being perceived as male.^37^

While some TGE people receive strong social support among their family members and communities, others feel isolated and alone during pregnancy.^38^ Feelings of loneliness are a consistent theme in the literature on TGE childbearing.^39^ At the same time, some trans men characterize pregnancy as a wonderful experience: “Pregnancy and childbirth were very male experiences for me. When I birthed my children, I was born into fatherhood.”^40^ Some people describe how pregnancy aligned with their gender or made them feel even more masculine.^41^ Whether pregnancy was a positive or negative experience, it is clear that navigating gender identity while pregnant requires significant work.^42^

#### 3. Pregnancy and Transition

Relatedly, research suggests there is wide variation in decision-making about pregnancy and transition. In a 2017 qualitative study on trans male pregnancy experiences, respondents reported becoming pregnant at different stages of social transition — whether before transitioning socially, while living part-time as male, or after a long period of living as male.^43^ Some deferred taking testosterone until after their childbearing was complete.^44^ Others began their medical transition, including testosterone, knowing (and fearing) that it might negatively impact their fertility but choosing to prioritize transition.^45^ By contrast, some who started testosterone before becoming pregnant reported feeling confident it would not preclude a later pregnancy, based on the experiences of other trans men.^46^

Indeed, testosterone use is a major factor — and subject of misinformation — in trans pregnancy experiences. Historically, testosterone has been considered a form of birth control for trans men, and providers have typically counseled transitioning patients that testosterone use will preclude pregnancy. Indeed, one study reported that 16% of trans men used testosterone as contraception and 5.5% reported than healthcare providers had advised them to do so.^47^ However, research on conception experiences shows this clinical advice is inaccurate, even for patients who are amenorrheic, or not menstruating.^48^ In a 2014 survey of transgender men, 61% reported using testosterone prior to pregnancy and among those, 24% had an unplanned pregnancy and 72% conceived within six months of trying to conceive.^49^ Of prior testosterone users, 80% resumed menses within six months of stopping the hormones.^50^ A 2021 study found a much lower rate of pregnancy among people who had used testosterone (3%) but a comparable percentage of people who ceased testosterone use to become pregnant (68% versus 73%).^51^ Testosterone does have teratogenic effects in pregnancy, posing a risk of abnormal urogenital development in female fetuses.^52^ This risk underscores the importance of accurate and comprehensive counseling about the impact of testosterone on fertility, not only to inform TGE people contemplating medical transition that testosterone use will not necessarily foreclose the ability to become pregnant in the future but also to reduce reliance on testosterone as contraception and the resulting risk of unintended pregnancy while taking testosterone.

Research is minimal on TGE people who have had gender-affirming surgery prior to (or after) pregnancy and childbirth. Certain surgeries would prevent pregnancy, such as hysterectomy or bilateral oophorectomy, but other gender-affirming surgeries do not. In the 2017 qualitative study, no participants had undergone genital surgery, but some had obtained chest surgery prior to pregnancy.^53^ More research is needed on the relationship between pregnancy and gender-affirming surgery. For example, it is unclear exactly how surgeries like metoidioplasty, scrotoplasty, or phalloplasty affect prognosis for vaginal delivery, leaving TGE people and their providers without important guidance for decision-making about medical transition.^54^

Furthermore, the diversity of experiences reported by TGE people related to pregnancy and transition has important mental health implications. While some people experience pregnancy as enjoyable and report no adverse effects from testosterone cessation, others struggle with the hormonal variability that results from tapering testosterone and from the discordance between their gender identity and the experience of being pregnant. For others, delaying medical transition due to concern about a possible impact on future pregnancy may harm the emotional and psychological health of someone experiencing dysphoria. The risk of adverse mental health effects among TGE people who wish to bear children — whether or not they have begun their transition—supports the need for more research, better counseling, and more support for TGE people navigating pregnancy and gender transition.

#### 4. Delivery Methods and Outcomes

Another area where TGE people report a wide variety of experiences is how they deliver their babies. Some research indicates a preference for cesarean delivery over vaginal birth among certain TGE people, despite the possible risks associated with surgery.^55^ For example, in the 2014 study, 36% of trans men who had used testosterone prior to pregnancy delivered by cesarean, compared with 19% of respondents who had not used testosterone, and among prior testosterone users, 33% reported requesting cesarean delivery while 0% of the cesareans among non-testosterone users were at their request.^56^ (Among the general public, 31.8% of pregnant people deliver by cesarean; the American College of Obstetricians and Gynecologists estimates that 2.5% of cesareans are the result of maternal request, although this phenomenon is not well understood.^57^) The overall percentage of pregnant men, regardless of testosterone use, who delivered by cesarean was slightly slower — 30% — and only 25% of the cesareans were at the pregnant person’s request.^58^ The preference for cesarean delivery reported by study participants might reflect fears about transphobia and mistreatment in the delivery suite or complex feelings about one’s genitalia that make vaginal delivery anxiety-producing or otherwise undesirable.^59^ More research on delivery method with larger sample sizes would be useful for shedding further light on the fears and motivations of TGE people during labor and delivery, as well as understanding the barriers to safe and non-traumatic birth experiences.

At the same time, research also suggests that a disproportionate number of TGE people seek care from a midwife instead of a physician and decide to give birth in a community setting — at home or in a freestanding birth center — rather than a hospital. In the 2014 study, 46% of participants obtained prenatal care from a midwife (including both in hospitals and community settings), 17% gave birth at home, and an additional 5% delivered in a birth center.^60^ Among the general population of birthing people, 98.4% deliver in a hospital, with only 0.99% giving birth at home and 0.52% delivering in a freestanding birth center.^61^ Only 8.7% of hospital births are attended by midwives; even when combined with midwife-attended community births, the proportion of birthing people generally who have midwife-attended births is significantly smaller than the proportion of TGE people who seek midwifery care.^62^

As discussed further below, the emphasis placed on individualized and holistic care under the midwifery model may be particularly appealing to TGE people. In addition, TGE people may disproportionately favor community birth settings because they are more likely to know who will be present during the birth and be able to screen for supportive, gender-affirming care providers in advance — as opposed to hospital settings, with their regular shift changes, the rotating on-call policies of OB/GYN practices, and (in academic medical centers) the likely presence of trainees. Interestingly, however, the non-interventionist approach of midwifery contrasts with the preference for cesarean delivery discussed previously, which illustrates how much diversity exists among the TGE childbearing population regarding what conditions feel optimal for achieving healthy pregnancy and safe delivery.

Turning to the potential impact of gender-affirming hormones on the health of childbearing TGE people and their babies, there are insufficient data to assess the potential impact of testosterone use on pregnancy, delivery, and birth outcomes.^63^ The 2014 survey contained no reports of anemia among any participants with prior testosterone use.^64^ Some authors have extrapolated from research on cisgender women finding an association between elevated endogenous androgen levels and reduced birth weight, but others have cautioned against relying on data about cisgender women to understand TGE populations who have used testosterone, and existing studies are too small to draw conclusions on this point.^65^ Existing research also does not distinguish between individuals who receive appropriate doses of testosterone and those who are inadequately maintained.^66^ Finally, there are no reliable data on the incidence of complications such as gestational diabetes or hypertension among TGE people, with or without testosterone use.

#### 5. Lactation and Chestfeeding

A final area of wide variation among childbearing TGE people is their experience with lactation and chestfeeding. Some TGE people choose to chestfeed their infants, regardless of whether they had chest surgery prior to pregnancy; in fact, the 2014 study reported that 51% of trans men chose to chestfeed their infants.^67^ In the 2017 study, some participants with prior chest surgery produced sufficient milk to feed their infants for more than six months, while others produced enough milk to meet their infants’ needs partially while supplementing with formula by bottle or at the chest using a supplementation system.^68^ Other participants experienced some chest swelling but did not lactate, and some respondents experienced neither.^69^ Ability to lactate and the quantity of milk produced may depend on the particular surgical approach employed, which underscores the importance of clinicians discussing fertility intentions with their patients before surgery.^70^ In addition, research suggests that testosterone suppresses milk supply, leading providers to recommend that testosterone resumption be avoided during the initiation of lactation, at least until milk supply is established.^71^

TGE people report a range of postpartum experiences that may lead to people valuing the benefits of chestfeeding differently. For some, a delay in resuming testosterone use prolongs the dysphoric experience of pregnancy and negatively impacts postpartum adjustment, including postpartum mental health more generally.^72^ Such experiences might prompt early cessation of chestfeeding, while others choose to bear the risk of a potentially negative impact of testosterone on nursing infants in favor of securing the benefits of human milk for the child and of testosterone resumption for the parent.^73^ Some research suggests that testosterone has low secretion into human milk and is unlikely to have an adverse effect on the infant if used after establishment of milk supply; this is yet another area where additional research would help postpartum TGE people make informed decisions.^74^ At the same time, other TGE people report that chestfeeding is a gratifying experience, enabling bonding with their infant and the productive use of body parts that had previously felt alienating.^75^

### B. Erasure of TGE Patients From Perinatal Care Systems

A second important theme that emerges from the research on TGE childbearing experiences is the difficulty patients have being seen and understood for who they are. Study participants report various ways they are excluded from spaces associated with pregnant women and prevented from accessing care as pregnant men or nonbinary people. Such erasure occurs at both the individual and institutional levels, beginning at the pre-conception stage and continuing through postpartum care.

The erasure of TGE experiences from reproductive health care means that many patients do not receive critical information from their health care providers about their fertility. Providers may assume incorrectly that someone who was assigned female at birth and no longer identifies as female is not interested in childbearing, thus foregoing counseling on fertility preservation before transition, general advice about pre-conception health, and specific advice about conception and health in early pregnancy, such as the need for folic acid supplementation to minimize risk of fetal neural tube defects. The erasure of TGE experiences from pre-conception and fertility-related care operates in a variety of ways, often contingent on the specific gender identity or transitioning process of an individual patient. For example, some providers assume that patients taking testosterone cannot get pregnant, leading to unintended pregnancy among some TGE people who mistakenly believe testosterone was adequate contraception. Other providers assume that patients taking testosterone do not wish to be pregnant, believing medical transition and pregnancy to be mutually exclusive and thus failing to help their patients develop a plan for safe cessation of testosterone before trying to conceive. Still other providers do not perceive the various reasons why a patient might taper testosterone use, such as cost barriers that may lead someone to stop testosterone temporarily or fluidity in gender identity that causes a patient to vary their testosterone use, which means such providers do not provide adequate counseling to enable or avoid conception.^76^

Failing to recognize the possibility of — and desire for — conception among TGE patients not only results in a disproportionately high unintended pregnancy rate, as discussed previously, but the fertility preservation rates for this population are low. Approximately 5-15% of transitioning adults and 2.8% of transitioning adolescents seek care related to fertility preservation.^77^ Certainly, the high cost of fertility preservation techniques, the invasiveness of the procedures, and the need to temporarily stop hormone therapy while undergoing treatment (if testosterone use has already commenced) all contribute to the low rates at which TGE people take advantage of fertility preservation.^78^ But the failure of providers to perceive that fertility counseling is appropriate for their TGE patients contemplating transition leaves a significant number of people with fewer childbearing options. Indeed, 38% of transgender men and 51% of transgender women report they would have considered gamete cryopreservation if they had been counseled about its availability before medical transition.^79^

Research on TGE childbearing experiences highlights various ways that people have to conceal their identities — or allow their gender identity to be erased — in order to gain access to spaces where health care and other pregnancy-related services are provided. Some trans men report pretending to be women in order to enable access to donor sperm through sperm banks and fertility clinics, attempting to avoid transphobic discrimination by these private entities that control access to necessary gametes.^80^ Trans men also report being unable to obtain appointments for prenatal care and pregnancy-related testing because the electronic information systems used by medical practices do not allow patients classified as male with their health insurance companies to schedule appointments for pregnancy-related services, as those are understood to be used by female patients only.^81^ Once patients are able to secure assistance overriding the system to schedule an appointment, those same record-keeping systems often do not differentiate between legal name and the name used by patients, which further obscures the pregnant patient’s gender identity from the people responsible for providing their care.^82^ TGE patients also report discomfort in OB/GYN waiting rooms and doctors’ offices where the bathrooms, decorations, and informational literature make clear that the space is designed only for female patients to use.^83^

The literature on TGE childbearing experiences is replete with examples of how patients’ gender identity is ignored or erased during labor and delivery by providers who misgender and deadname patients, address and discuss the patient as “mom,” and refer to body parts using language with which the patient has indicated discomfort.^84^ The inability of electronic records systems to recognize a pregnant man may result in denial of care during labor, such as in the case of a study participant who was denied an epidural because the hospital’s records system would not allow a male patient to obtain the fetal monitoring necessary for the epidural.^85^ Once the baby is born, TGE patients find it difficult — or impossible — to be named as the baby’s father (or parent) on the birth certificate; in many jurisdictions, this requires extensive legal effort or a separate adoption proceeding of one’s own children.^86^

In addition, as the new parent recovers from childbirth, postpartum depression and other perinatal mood disorders may go unaddressed because providers do not recognize the particular mental health challenges of their TGE patients. Although the baseline rates of depression and suicide are higher among transgender individuals than the general population, many TGE patients report receiving no counseling about the risk of postpartum depression and may have difficulty distinguishing between depression and the impact of hormonal changes related to testosterone resumption.^87^ Furthermore, the Edinburgh Postnatal Depression Scale used to screen for postpartum depression is not designed to account for gender dysphoria among TGE patients, which increases the difficulty of diagnosing postpartum mental health complications.^88^ By failing to account for TGE people getting pregnant and giving birth, the systems through which perinatal care is delivered and the professionals responsible for providing that care contribute to the erasure of TGE people from their own childbearing experiences.

The omission of childbearing TGE people from research on reproductive health, their exclusion from clinical settings, and their neglect in the training and skills of health care providers are forms of systemic bias. Provider lack of familiarity with trans experiences and with the biomedical aspects of medical transition reinforce each other to erase TGE people from the reproductive health care landscape.

The erasure of TGE people from perinatal health systems is enabled and exacerbated by the minimal research available on the reproductive healthcare needs and experiences of this population. Unlike Australia, which tracks the number of trans men who give birth each year through its national insurance program, the United States lacks data on the prevalence of pregnancy in trans men, as well as nonbinary and other gender-expansive people.^89^ As highlighted in previous sections, there is a general lack of reliable data about the impact of testosterone on reproduction, barriers to and methods of conception, pregnancy complications and outcomes, perinatal mental health impacts, and postpartum care and lactation.^90^ Furthermore, the limited research that does exist focuses on TGE people who have conceived and given birth, not TGE people who were unable to conceive or who conceived but whose pregnancies ended in miscarriage, abortion, or stillbirth.^91^ Existing studies also include few — if any — participants who identify as racial or ethnic minorities, live on lower incomes, have less formal education, or have a disability.

The lack of data and clinical guidance for perinatal care of TGE people contributes to provider reluctance to take on TGE patients. Some providers are unwilling to educate themselves about care needs that are unique to TGE patients, and the limited research literature compounds provider fear of “getting it wrong, in addition to feeling uncomfortable.”^92^ One study participant noted that when TGE patients do find providers willing to care for them, it is important that those providers “differentiat[e] between ‘I don’t know’ and ‘science doesn’t know,’” so that patients do not rely on guesswork by providers but instead can weigh various risks and benefits informed by data on the topic, however limited.^93^ Ultimately, the gaps in knowledge about TGE childbearing leave people in “informational isolation,” relying on anecdotes and personal networks for examples of transmasculine pregnancy and information about how to navigate testosterone use, conception, prenatal care, and lactation on their own.^94^

The omission of childbearing TGE people from research on reproductive health, their exclusion from clinical settings, and their neglect in the training and skills of health care providers are forms of systemic bias. Provider lack of familiarity with trans experiences and with the biomedical aspects of medical transition reinforce each other to erase TGE people from the reproductive health care landscape. As one commentator notes, these “combined conditions convey[] the message that [TGE] lives could not exist within the system, and their identities did not matter.”^95^ This erasure from the perinatal health care system reflects the broader phenomenon of societal erasure. A pregnant man is “unintelligible” not only to his health care providers but also among the broader community, leaving him without needed health care services as well as social support during pregnancy and childbirth.^96^

### C. Harmful Gender Norms in Childbirth-Related Care

The erasure of TGE people from perinatal health care discussed in the previous section is linked to gender’s central role in the cultural meaning of pregnancy and childbearing. Entrenched gender norms not only shape what questions researchers ask, but they influence the training of providers, the design of clinical spaces and record-keeping systems, and the provision of care itself. Research on TGE childbearing both confirms and further illuminates how gender stereotypes associated with pregnancy impact the quality of care TGE people receive. In many respects, society’s insistence that pregnancy and birth are (cisgender) female-identified experiences inflicts harm on pregnant TGE people because it constructs them as a threatening challenge to the current system.^97^

The strong association between womanhood and pregnancy shapes provider expectations regarding who gets pregnant and who wants to become pregnant.^98^ As discussed above, providers fail to anticipate that their TGE patients may wish to bear children and need information about fertility preservation, testosterone use and conception, and contraception — leaving patients to pursue self-help or experience adverse health consequences due to ignorance of their own reproductive health. Provider blindness to potential childbearing goals of their TGE patients can also lead to recommendations for gender-affirming surgery that the patient may not need or want.^99^ Confronting the pervasive belief that pregnancy is a female experience, some trans men actively challenge the perception of pregnancy and transition as incompatible by educating their providers about trans-specific reproductive health care or by living outwardly as pregnant men.

Other trans men report feeling like they had to choose between receiving gender-affirming treatment or fertility-preserving care because their providers believe that only cisgender women desire pregnancy and thus someone who wants to be pregnant in the future is not an appropriate candidate for transition.^100^ They hide their childbearing goals from providers of gender-affirming care in order to receive the hormones or surgical treatment they need.^101^ Though understandable under the circumstances, withholding relevant information from one’s healthcare provider interferes with the provision of appropriate medical care. That some people feel the need to employ this strategy to secure gender-affirming care reflects the extent to which reproductive desires are erased for trans people, even among providers who may be perceived as trans allies. Insurance systems further exacerbate the binary gender norms that require TGE people to choose between important forms of health care. For example, knowing that insurance will cover either gender-affirming chest surgery or pregnancy and childbirth may force a patient to time decisions about major life changes based on availability of insurance reimbursement.^102^

Powerful gender norms in perinatal health care lead to other forms of provider bias against pregnant TGE patients. Some women’s health providers refuse to treat trans male patients altogether.^103^ When TGE people are able to find care, they report a range of ways their health care providers lack cultural competency: using the wrong titles and pronouns; calling patients by their given names instead of the name indicated by the patient; ignoring information provided about gender on intake forms; assumptions about genitalia based on the patient’s name or physical appearance; assumptions about the patient’s relationship with their body; and confusion between sexual orientation and gender identity.^104^ Multiple studies contained findings that illustrated how people default to female-oriented language when discussing pregnancy or providing childbirth-related care. For example, people who had transitioned before pregnancy and were living outwardly as men reported that even trans friends sometimes slipped into using female pronouns and gendered language to address them while pregnant.^105^ Even the default language used to describe the care (“maternity care”) and the location in a hospital where it is provided (“maternity ward”) reflects how deeply gendered this form of health care is.

The disciplining power of gender in perinatal health care results in more than microaggressions. Some TGE people report being tokenized and objectified by their providers, receiving unnecessary physical exams and being asked questions that felt “prurient, exotifying, voyeuristic, and superfluous to the patient’s care.”^106^ They are mocked, treated rudely, and denied particular forms of care, such as lactation support.^107^ Others describe how providers pathologize them for deviating from dominant gender norms associated with childbearing. Providers have reported their patients to the child welfare authorities and patients have been investigated as unfit parents simply for being trans and pregnant.^108^ One respondent reported being required to undergo a psychological consultation before the physician would sign paperwork necessary to obtain donor sperm.^109^ Fear of mistreatment, along with the high degree of stigmatization experienced by patients who transgress the gender norms of pregnancy, lead some transgender men to avoid or delay prenatal care, or to withhold relevant information from their care providers. The fact that these forms of provider bias occur against a backdrop of individual and structural violence aimed at trans and other gender diverse people in the broader society compounds the adverse impact of such bias on patients’ health.^110^

As noted above, TGE people are significantly more likely than cisgender women to seek midwifery care and to give birth in community settings. It is likely that fear of transphobia and gender-based discrimination motivates some people to avoid hospital settings, where birthing patients are required to interact with a greater number of medical staff, as well as other patients, all of whom might pose a risk to TGE people’s feelings of safety during labor, delivery, and postpartum care. TGE people giving birth in hospitals bear the burden of repeated coming out and continual provider education every time there is a shift change or new medical personnel enter the room. Furthermore, physical touch and the presence of strangers in the delivery room may produce anxiety in TGE patients, making the midwifery model’s approach to individualized care particularly appealing to some pregnant TGE people.^111^ Community birth also enables TGE patients to avoid gendered spaces within the hospital, including the potential sharing of a postpartum room with a cisgender woman.

Ultimately, the strong association of childbearing with female-identified patients and femininity can mean that childbirth is fraught terrain for TGE people to navigate, with risks of various kinds of harm inflicted by transphobic providers, along with well-meaning providers whose thinking is nevertheless so influenced by gender norms that they misunderstand their TGE patients’ experiences and health care needs. After giving birth, TGE patients not only experience the typical challenges of postpartum recovery but may also bear the burden of healing from emotional and psychological harm that results from having to navigate a highly gendered perinatal care system.

## III. Reconceiving Health Care for Childbearing TGE People

Although more research on TGE childbearing experiences is needed, the existing literature contains sufficient findings to conclude there are numerous barriers preventing TGE people from accessing safe, gender-affirming reproductive health care. With increased public attention on the maternal mortality crisis in the United States and calls for interventions to reduce the number of women — particularly Black women — dying in childbirth, there are opportunities to make the healthcare system responsive to the needs of pregnant TGE people while also reducing adverse health outcomes more generally.^112^ To be effective, reform must address the harmful impact of rigid gender norms on the provision of perinatal health care. It is not sufficient to train providers on providing respectful care to individual TGE patients if the underlying systems remain blind to TGE people when they need reproductive health care related to childbirth. In particular, the Midwives Model of Care offers an approach to individualized, holistic, patient-centered care that could help achieve the transformation necessary to meet the perinatal health care needs of TGE patients.

### A. The Need for Systemic Change

One insight from the research on TGE childbearing discussed previously is that there is wide variation in how TGE people experience pregnancy and childbirth, which suggests that the best practice for one is not necessarily the best practice for all. A series of case studies in the literature on trans health care illustrates this point and underscores the necessity for a multiplicity of approaches to meeting the needs of gender diverse patients. First, a trans man admitted to the hospital for bariatric surgery recounted the harm caused when he was required under hospital protocols to take a pregnancy test before receiving care.^113^ The patient experienced this requirement as a humiliating denial of his gender identity and a barrier to receiving health care. Elsewhere, a trans man presented at the hospital’s emergency department with abdominal pain and high blood pressure.^114^ He identified himself as a trans man with hypertension who had ceased taking testosterone and blood pressure medications because he had recently lost health insurance coverage.^115^ Staff did not screen for pregnancy complications, which resulted in delayed recognition that the man was pregnant and had developed preeclampsia, resulting in an emergency cesarean and stillbirth.^116^

Healthcare providers may consider these cases together and perceive a “damned if you do, damned if you don’t” quality to them, as recognizing a trans patient’s capacity for pregnancy in one situation was an affront to their identity and failure to recognize a trans patient’s actual pregnancy in another circumstance resulted in tragic loss and needless suffering. The ethical challenges posed by such scenarios may prompt some providers to disengage and continue to ignore the perinatal healthcare needs of TGE populations — an option made possible in part by the relative invisibility of such patients within female-oriented perinatal health care spaces. It may feel too difficult to understand the varying care needs of TGE patients and figure out how to provide quality care, but it does not have to be this way, especially if the healthcare system were reimagined to account for gender diverse patients. Record-keeping systems could be adjusted to capture relevant information about a patient’s gender identity and transition history without forcing patients to fit themselves into preexisting categories. Patient intake could include talking to patients about what body parts they have, how the patients refer to those parts, and what meaning patients assign to body parts encompassed in the reproductive system. And electronic records, billing software, and insurance reimbursement systems could be reconfigured to diagnose, record, and reimburse for care in a way that does not depend on rigid gender categories and assumptions.

Clearly, there are significant gaps in provider education and training about the TGE patient population, but various resources exist for providers who want to learn about the medical needs of their TGE patients, including aspects of reproductive health care that are specific to TGE patients.^117^ Experts have issued guidance for providing gender-affirming care, recommendations that, if followed, would make a meaningful difference in the perinatal health care experiences of TGE patients. For example, use of the appropriate name and pronouns is necessary for patients to feel safe; office staff should be trained not to make assumptions about a patient’s gender based on the sound of someone’s voice over the phone or when presenting at check-in; hospital staff should limit who enters the room during labor and delivery; providers should normalize trans pregnancy, avoiding intrusive questions or exotifying remarks; providers should not expect patients to teach them how to provide appropriate care but should listen when patients do share relevant information; providers should be able to differentiate between what they personally do not know and what science does not know about TGE childbearing; providers should use abdominal over transvaginal ultrasound whenever possible; patients seeking transition-related care should be counseled about their reproductive options, including fertility preservation, the impact of testosterone on conception, and the effects of chest surgery on lactation; and patients need counseling about the impact on mental health of ceasing testosterone for pregnancy, navigating pregnancy in a transphobic world, and postpartum adjustment.^118^

These types of changes in clinical practice are necessary for providing gender-affirming care to TGE patients, but they are not sufficient to accomplish systemic change. Policies and protocols are important tools in shifting behavior and helping providers establish new habits in their clinical practice. But it is also essential to change provider mindset — and the orientation of the system more broadly — so that gender is not the lens through which pregnant patients, and all TGE patients, are seen and understood. Indeed, the erasure of TGE people’s reproductive capacities occurs across the healthcare system, not simply in perinatal health care. One study participant conveyed the type of burdens TGE people bear when seeking health care, using the example of a finger wound that had persisted for over six months.^119^ The individual sought care for the wound, disclosing to the doctor that they were trans and currently chestfeeding, and ultimately received no treatment for the wound but was asked inappropriate questions about breastfeeding while trans.^120^ When the patient saw a second doctor, they did not disclose that they were trans or chestfeeding, and were prescribed medication that was unsafe for a nursing baby.^121^ Ultimately, it took five different doctors to secure appropriate treatment for the finger wound, requiring a degree of time investment, financial cost, and emotional burden that leaves TGE patients depleted and acts as a deterrent to seeking care in the future.^122^

The healthcare system’s failure to account for or acknowledge gender diversity leaves many gaps in care, such as whether breast cancer screening recommendations for trans women should be based on chronological age or length of exposure to exogenous estrogen, when and how to conduct prostate exams for trans women, and what type of chest/breast cancer screening is appropriate after chest surgery. Reliance on gender to establish care guidelines can also result in unnecessary treatment and painful reminders of prior health complications, such as for the cisgender woman who receives automated reminders from her insurer based on her female status to schedule a pap smear, despite the fact that she underwent a complete hysterectomy and is not recommended to continue having pap smears. Moving away from the use of gender to organize care recommendations and instead using the existence of particular body parts to categorize patient needs is an important step in achieving a system where all individual patients’ healthcare needs are recognized on their own terms, thus avoiding erasure and other burdens that disproportionately fall on TGE patients.

### B. Accounting for the Range of TGE Patient Experiences: Learning From Midwives

As providers learn to resist one-size-fits-all thinking and educate themselves to become more competent providers of perinatal health care to TGE patients, they should look to the Midwives Model of Care for a framework that is well-suited to accommodate the wide variation in TGE patient needs. The Midwives Model of Care includes “monitoring the physical, psychological and social well-being of the [birthing person] throughout the childbearing cycle[;] providing the mother/birthing parent with individualized education, counseling, and prenatal care, continuous hands-on assistance during labor and delivery and postpartum support[;] minimizing technological interventions[;] and identifying and referring women/birthing people who require obstetrical attention.”^123^ Midwives prioritize holistic, patient-centered, non-interventionist care. They care for people experiencing low-risk pregnancies and have robust risk assessment procedures for determining whether someone has pregnancy risk factors that make them a better candidate for physician care.^124^ Some midwives also provide pre-conception counseling and fertility care, assisting with insemination outside of a fertility clinic setting.^125^

In the United States, midwives have different credentials and different licensure statuses depending on the jurisdictions where they live and work — an unnecessarily complicated patchwork of legal recognition that is the product of the medical profession’s efforts, starting in the nineteenth century, to marginalize midwives and exercise physician control over childbirth.^126^ The two most common forms of midwives are certified nurse midwives (CNMs) and certified professional midwives (CPMs).^127^ CNMs obtain nursing training before specializing in childbirth-related care and typically practice in hospitals alongside — and often under the supervision of — physicians, despite the fact that they are fully trained experts in low-risk childbirth.^128^ CPMs obtain their midwifery training directly without first training as a nurse and attend births in community settings, either at home or in freestanding birth centers.^129^ CPMs generally face more burdensome regulatory climates than CNMs; for example, while CNMs are licensed in all fifty states and other territories, CPMs are currently licensed to practice in only 37 states and DC.^130^ Both types of midwives also face a variety of other restrictions on their ability to practice to the full extent of their education and training, which inhibits growth of the profession, impedes integration of midwives into the broader perinatal healthcare system, and limits access to midwives for pregnant people who seek this type of care — all of which leads to worse health outcomes for birthing people and their babies.^131^

Research shows that midwifery care is safe, though some physicians continue to criticize midwifery and discourage its use. Such opposition to midwifery may be the legacy of racist, anti-immigrant propaganda campaigns launched by early physicians eager to assert control over the market for childbirth services or reflect the perception that midwives pose a threat to physicians’ livelihood.^132^ Whatever the cause, disinformation about midwifery has contributed to the suppression of midwives in the United States—in contrast to many other developed nations where midwives play a central role in perinatal health care — and has confused the public about whether midwifery care is a reasonable option. In fact, not only is midwifery care safe, but when compared with physician-led care, midwifery is also associated with better outcomes on several perinatal health measures, fewer procedures during labor, and increased satisfaction of patients.^133^

Pregnant people who choose midwifery care may do so for a variety of reasons. Some are drawn to the holistic, individualized approach to care, with prenatal appointments that last up to an hour (as opposed to 5-15 minutes for typical obstetrics practices), attention to the psychosocial aspects of pregnancy and the transition to parenthood, and inclusion of family or other support people in the care. Midwifery practices tend to be smaller than obstetrics practices, enabling more relationship-building between the pregnant person and their provider(s). Some seek midwifery care due to its non-interventionist philosophy and emphasis on robust informed consent processes, where providers are not bound by hospital protocols that may be inappropriate for particular patients (at least for midwives practicing in community settings) and pregnant people take a more active role in childbirth-related decision-making than is contemplated or encouraged in many hospital practices. Still others seek community-based midwifery care because they have had negative experiences in medical settings, whether in previous pregnancies or related to other health care needs. In particular, a growing number of Black women who face bias in mainstream medicine, suffer mistreatment by their doctors, or have experienced trauma during previous births are seeking midwifery care for subsequent pregnancies.^134^

For all of these reasons, midwifery care is an attractive option for many TGE people. Starting with fertility care, some midwives offer pre-conception counseling and intrauterine insemination (IUI) services, a common method of achieving pregnancy for people using donor sperm or those who do not wish to engage in penetrative sex in order to conceive.^135^ Indeed, some midwifery practices cater explicitly to queer and trans people, offering gender-affirming fertility and pregnancy care that is attuned to the needs of LGBTQ+ prospective parents.^136^ Access to such care can enable TGE people who experience social infertility due to their relationship status or choice of partner to bypass invasive medical testing used to diagnose medical infertility in cisgender heterosexual patients.^137^ Midwife-led fertility care enables TGE people to avoid highly-gendered fertility clinics and receive lower-cost, personalized, non-pathologizing care at home.

In addition, the smaller size of midwifery practices and closer relationships between the pregnant person and provider enable TGE people to determine more accurately whether their midwife understands their needs and will provide gender-affirming care throughout pregnancy and childbirth, rather than living with uncertainty about whether the medical staff on call when labor begins will be transphobic or supportive. The patient-centered focus of midwifery care provides more opportunities for individualized care plans, including frequency (or lack of) cervical checks during labor, use of preferred language for reproductive organs and other body parts, and creating the kind of environment that will make the birthing person feel safe. And the commitment to informed consent as a continuing process throughout labor and delivery gives people who experience gender dysphoria, have a history of trauma, or otherwise have a fraught relationship with their bodies the opportunity to control when and how they are touched during childbirth. This approach stands in contrast to the norms that govern obstetrics care in some hospitals, where patients are deemed to have given implied consent to certain forms of touch — and even some medical interventions — simply by having come to the hospital for admission while in labor.^138^

Given the values and practices that characterize midwifery care, it is not surprising that a disproportionate number of TGE people perceive meaningful differences between midwife-led care and physician-led care, choosing midwives and opting to deliver in community settings more often than cisgender women. Indeed, the American College of Nurse-Midwives (ACNM), the national professional association for CNMs, has issued a position statement on providing gender-affirming care for TGE people in which it explained that “the midwifery model of care is particularly well suited to assume this role because of its respect for autonomy, self-determination, and shared decision-making.”^139^ In an update to clinical guidelines issued in 2021, ACNM also stated that provision of gender-affirming hormone therapy falls within the scope of practice for CNMs and that such care is now included in many midwifery education programs.^140^

Although the promotion of midwifery has been recognized as one meaningful approach to improving health outcomes, midwifery is not a panacea for all that ails modern maternity care in the United States. Nor is midwifery care appropriate for all pregnant TGE people. As discussed previously, research suggests some trans men may actively prefer cesarean delivery over vaginal birth as a strategy for coping with gender dysphoria, which would make hospital birth with an obstetrician feel like a safer, more gender-affirming choice. In addition, it is possible that pregnant TGE people could be at disproportionate risk of certain pregnancy complications, such as preeclampsia, due to the increased stress of navigating a transphobic world while pregnant — similar to findings that suggest stress caused by racism could help explain why Black women are at greater risk of certain adverse perinatal health outcomes, regardless of education or socioeconomic status.^141^ As researchers learn more about the pregnancy outcomes of TGE people, recommendations about where and with whom TGE people should give birth must take a holistic view of all the factors contributing to pregnant TGE people feeling safe during childbirth.

In addition, not all midwives are appropriate providers for TGE people. Midwives are exposed to the same dominant cultural norms as the general public and may express transphobic views or adhere to stereotypical gender norms that exclude TGE people. Further, some midwives hold an essentialized view of gender and pregnancy, understanding themselves to provide an alternative to medicalized hospital birth and its interference with feminine power expressed during childbirth. In recent years, the midwifery community in the United States has grappled with public and private debates about the use of gendered language, as well as whether and how to care for pregnant TGE people.^142^ While some midwives remain deeply uncomfortable with — or hostile to — the idea of a pregnant man, others have been galvanized to educate themselves about providing gender-affirming midwifery care and to support LGBTQ+ people on the path to midwifery in order to grow the proportion of the midwifery workforce who are themselves gender-diverse or are allies equipped to provide competent care.^143^

Ultimately, the elimination of burdensome, non-evidence based regulatory restrictions on midwives would be an important step towards expanding access to midwifery care for TGE people who want it, especially in parts of the country where options for gender-affirming perinatal care are otherwise limited.^144^ Furthermore, although midwifery care is not appropriate or accessible to all childbearing TGE people, lessons from the midwifery model about patient-centered care and informed consent would benefit the healthcare system more broadly and ensure that all birthing people receive safe, high-quality, respectful perinatal care.

## IV. Conclusion

The need for more research on the childbearing experiences of TGE populations is urgent and extensive. Although the existing literature provides meaningful insight about how the perinatal healthcare system in the United States does not meet the needs of pregnant TGE people for safe, quality, gender-affirming care, it contains many gaps. These gaps leave pregnant people and their healthcare providers without useful guidance on critical issues like the impact of testosterone on conception and pregnancy outcomes or how to recognize and treat perinatal mood disorders in TGE patients. At the same time, the thin research on TGE childbearing experiences limits the effectiveness of efforts to promote systemic change in the provision of perinatal health care for the TGE population. Just as some trans men report that being legible to the public as a pregnant man is empowering, we need to make TGE childbearing experiences visible to stakeholders within the healthcare system in order to ensure that TGE patients receive care that recognizes their full humanity.

Other scholars have enumerated many topics related to TGE childbearing that require further study. In addition, future research should prioritize the decision-making of TGE people around care provider and birth setting to understand better why TGE people perceive midwifery care as desirable and pursue community birth with midwives at significantly higher rates than the general public.^145^ At the same time, given that a disproportionate number of TGE pregnant people seem to perceive cesarean surgery as the optimal way to deliver, research on this aspect of childbirth decision-making among TGE people would help to identify the motivations underlying cesarean preference, as well as the concerns and desires of TGE birthing people more generally. Given the discordance between midwifery preference and elective cesarean preference reported by TGE pregnant people, it would be useful for future research to explore any relationship between these findings in light of the possibility that greater access to individualized, gender-affirming, trauma-informed midwifery care would help more TGE people feel safe delivering vaginally, enabling them to forego the more invasive, more expensive, and riskier surgical route. Deeper insight into the motivations, goals, and fears of pregnant TGE people as they prepare to give birth may suggest how to replicate elsewhere in perinatal health care the positive experiences TGE people have with their midwives and also support arguments for greater integration of midwives into the healthcare system. Relatedly, research and advocacy to advance interprofessional collaboration among perinatal care providers, including those who work in different birth settings, should include the needs of TGE patients in exploring and promoting such models of care.^146^ Such changes would benefit the growing number of TGE people choosing to become pregnant and give birth, as well as the many cisgender people whose needs are not met by the current system.

## Note

The author has no conflicts of interest to disclose.
